# Prevalence Trends of Metabolic Syndrome among Korean Children and Adolescents from a Population-Based Cross-Sectional Survey

**DOI:** 10.3390/life12091404

**Published:** 2022-09-09

**Authors:** Ji Hyun Kim, Jung Sub Lim

**Affiliations:** 1 Department of Pediatrics, Dongguk University Ilsan Hospital, Goyang 10326, Korea; 2 Department of Pediatrics, Korea Cancer Center Hospital, 215 Gongneungdong, Nowon-gu, Seoul 01812, Korea

**Keywords:** syndrome, metabolic, youth, epidemiology, pediatric obesity

## Abstract

The prevalence of metabolic syndrome (MetS) is increasing worldwide. This study aimed to investigate the prevalence trend of metabolic syndrome among Korean adolescents and to examine the effect of changes in dietary components on metabolic syndrome components. It has used the data of children and adolescents (8718 subjects aged 10–18) from the National Health and Nutrition Survey IV-VII (KNHANES 2007–2018) to estimate the recent prevalence of MetS and identify related nutritional factors. The definition of MetS used modified NCEP-ATP III and IDF criteria. The prevalence of MetS among Korean adolescents in 2007–2018 was 4.6% using the modified NCEP-ATP III criteria, and the trend of MetS increased significantly (*p* trend = 0.02). In the overweight and obese groups, the risk of MetS increased 7.08 (95% CI, 5.19–9.79) and 27.13 (95% CI, 20.90–35.24) compared to the normal-weight group. During KNHANES IV-VII, overall caloric intake increased, carbohydrate and sodium intake decreased, but fat intake increased (KNHANE-IV; 21.3% to VII; 24.0%, *p* < 0.001). These fat intakes were significantly correlated with an increase in systolic blood pressure, fasting blood glucose, and waist circumference. The prevalence of MetS is also increasing in Korean adolescents, and changes in dietary habits are related. In the future, it is also necessary to study the relationship of MetS to lifestyle.

## 1. Introduction

Metabolic syndrome (MetS) is a cluster of abdominal obesity, increased blood pressure (BP), elevated glucose, and dyslipidemia [1,2]. MetS and insulin resistance syndrome, are major targets for preventing cardiovascular disease (CVD) and type 2 diabetes mellitus (T2DM), as both are associated with increased CVD and diabetes-related morbidity and mortality in both European and Asian populations [3,4,5]. Besides, adolescents with MetS show not only earlier development of T2DM and CVD in adolescents and mid-adults but also show a decline in quality of life [2]. Thus, early detection and appropriate intervention against MetS and its risk factors is critical to promoting current and future health in children and adolescents.

Current estimates indicate that the age-adjusted prevalence of the MetS among Korean adults was 28.9% without a significant change between 2008 and 2013. During those periods, smoking rate, sodium intake, and physical activity significantly decreased, while total calorie intake and intake of carbohydrate, fat, protein, and calcium significantly increased. In Korean adults, the odds ratio (ORs) of MetS in obesity was 6.7 [6].

Worldwide, the prevalence of obesity among children and adolescents has continued to increase substantially over the past decades [7]. The global age-standardized prevalence of obesity increased from 0.7% in 1975 to 5.6% in 2016 in girls, and from 0.9% to 7.8% in boys. The prevalence is characterized by a steep increase in developing countries, primarily East Asia [8,9]. In Korea, Park et al. reported a significant increasing trend of obesity, but MetS was stable in Korean adolescents from 1998 to 2005 [10]. Obesity is a discernible MetS risk factor. Therefore, it is likely that the prevalence of MetS phenotype has increased among Korean children and adolescents. 

Thus, the aims of this study were as follows: (1) to update the most recent prevalence of MetS and its components of Korean aged 10 to 18 years; (2) to investigate the trends of the prevalence of MetS and its components during 2007 and 2018; and (3) to evaluate the trends in overall dietary factors that might be related to any changes in MetS.

## 2. Materials and Methods

### 2.1. Subjects

The Korea National Health and Nutrition Examination Survey (KNHANES) IV to VII (2007–2018) data has been used in this study. KNHANES is an ongoing surveillance programme that was initiated to produce nationwide statistics regarding the health status, health behaviors, and food and nutrient consumption of the Korean population [11]. This survey is conducted periodically by the Korea Institute for Health and Social Affairs and the Korea Health Industry Development Institute, and includes nutritional surveys related to chronic diseases. Informed consent was obtained from all participants in the KNHANES. This study was approved by the Institutional Review Board of the Korea Disease Control and Prevention Agency (KDCA, formerly Korea Centers for Disease Control and Prevention). All subjects and their parents were interviewed at home after informed consent had been given and underwent various examinations. 

A total of 10,735 subjects (5670 male and 5065 female) aged 10.00 to 18.99 years were identified as potential subjects for this study. Those with incomplete data for a standardized physical examination, lab test, and who did not fast ≥8 h were excluded (*n* = 2244). Thus, the final analytical sample consisted of 8491 subjects (male = 4510). 

### 2.2. Measurements

The method of anthropometric and laboratory measurement was processed according to the KNHANES protocol. A detailed description of KNHANES’ survey planning and operation can be found on its website (http://knhanes.cdc.go.kr/, accessed on 1 July 2022) [11]. In brief, the health interview and health examination are performed by trained medical staff and interviewers. After the health interview and health examination surveys, dieticians visit the homes of participants for the nutrition survey. 

All anthropometric measurements were performed by well-trained examiners, who used a standard protocol for all four KNHANES cycles. Weight was determined to the nearest 0.1 kg on a medical scale (GL-6000-20, G-tech, Seoul, Korea); height was measured to the nearest 0.1 cm with a wall-mounted stadiometer (Seca 225, Seca, Hamburg, Germany). Body mass index (BMI) was calculated by dividing the weight (kg) by the height squared (m^2^). Blood pressure (BP) was measured with a mercury sphygmomanometer (Baumanometer sphygmomanometer; W.A. Baum Co Inc., Copiague, NY, USA, and Littmann Stethoscopes; 3M, Maplewood, MN, USA) according to protocol. 

Blood samples were taken by a skilled nurse after fasting for at least 8 h and transported daily to the Central Laboratory (NEODIN Medical Institute, Seoul, Korea). Fasting plasma glucose (FPG), triglyceride (TG), and high-density lipoprotein cholesterol (HDL-C) concentrations were measured by Hitachi Automatic Analyzer 7600 (Hitachi, Tokyo, Japan). 

Dietary intake was determined from a 24-h food recall administered by a trained dietary interviewer in the mobile examination center on the examination day. The equations for % of each nutrients were as follows: the % total energy from carbohydrates = (4 × grams of carbohydrates)/total calories; the % total energy from fats = (9 × grams of fat)/total calories; the % total energy from protein = (4 × grams of protein)/total calories.

### 2.3. Definition of the Metabolic Syndrome

National Cholesterol Education Program Adult Treatment Panel (NCEP-ATP) III definition and International Diabetes Federation (IDF) definition of MetS [12] has been used in this survey. To be classified as having MetS, this study employs the term as defined by modified NCEP-ATP III definition, with at least 3 of the following 5 risk factors: elevated FPG levels were defined as glucose levels ≥ 100 mg/dL. An elevated TG level was defined as a fasting TG level ≥ 110 mg/dL, and low HDL-C was defined as an HDL-C level < 40 mg/dL. Elevated blood pressure was defined as BP ≥ 90th percentile, or in receipt of treatment for hypertension. Central obesity was defined as a waist circumference ≥ 90th percentile. In MetS by IDF, at least 2 of the following 4 were risk factors for central obesity (a waist circumference ≥ 90th percentile, >16 years; male ≥ 90 cm. female ≥ 80 cm): elevated FPG (≥100 mg/dL), elevated TG (≥150 mg/dL), low HDL-C (male < 40 mg/dL, female < 50 mg/dL), elevated BP (a systolic BP ≥ 130 mmHg or a diastolic BP ≥ 85 mmHg, or in receipt of treatment for hypertension). 

The subjects were classified as obese (BMI ≥ 95th percentile), overweight (BMI ≥ 85th and <95th percentile), and normal weight (BMI < 85th percentile), based on 2017 Korean CDC growth charts and abdominal obesity was defined using the cut-off points (90th percentile for age and gender) [13]. The reference of BP is based on normal-weight Korean children and adolescents with the same sphygmomanometer [14]. 

### 2.4. Statistical Analysis 

Data relating to anthropometric measurements and other covariates were stratified by the KNHANES cycle (IV; 2007–2009, V; 2010–2012, VI; 2013–2015, and VII 2016–2018). Continuous variables were reported as mean ± standard deviation (SD), and categorical variables were reported as percentages. Differences among the KNHANES cycle were compared using ANOVA test and Chi-square test. Linear regression was used to estimate a means of quantitative measurements (etc.) across each KNHANES cycle as a continuous variable and assess for temporal linear trends. Logistic regression analyses using linear by linear results were used for predicting the prevalence trends of MetS and its components. The results were derived during logistic regression analysis by correcting the gender, age, and BMI z-score.

Fasting plasma TG data were log-transformed for regression analysis and back-transformed for data presentation. All statistical analyses were performed using SPSS 17.0 for Windows (SPSS Inc., Chicago, IL, USA). *p*-values < 0.05 were considered significant.

## 3. Results

### 3.1. Characteristics of the Study Subjects

Table 1 shows the characteristics of the study subjects: anthropometric, biochemical parameters, and nutrient factors, stratified according to the KNHANES cycle (2007–2018). The age at the time of the survey of the study subjects was 13.8 ± 2.5 years, and males composes 53.1%. The prevalence of obesity (BMI ≥ 95th percentile) was increased according to the KNHANES cycle (*p* trends = 0.001). Waist circumference was increased, and HDL-C was increased. 

### 3.2. Trends and Prevalence of MetS and Its Components during in the KNHANE IV (2007–2009) to VII (2016–2018)

**Figure 1:**
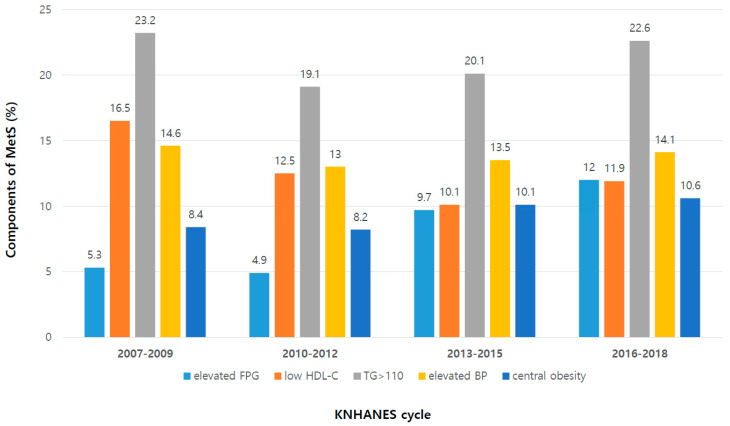
Changes in the composition of the five components of metabolic syndrome according to KNHANES cycles. Fasting blood sugar and central obesity increase, but low HDL shows a decreasing trend. The definition of metabolic syndrome used modified NCEP-ATP III. KNHANES, Korea National Health and Nutrition Examination Survey.

Table 2 shows the prevalence of MetS and its five components. The overall prevalence of modified NCEP-ATP III MetS in the KNHANES IV–VII was 4.6%. This overall prevalence varied by gender (males 5.2%, females 3.9%, *p* = 0.003). During the KNHANES cycles, the prevalence of MetS significantly increased during the period observed (*p* trends = 0.020). The prevalence of MetS was 4.5%, 3.9%, 4.1%, and 6.2% in KNHANES IV, V, VI, and VII, respectively. In KNHANES IV, the most prevalent component of MetS was elevated TG (23.2%), followed by low HDL-C (16.5%), elevated BP (14.6%), abdominal obesity (8.4%), and elevated FPG (5.3%). Meanwhile, the overall prevalence of IDF MetS in the KNHANES IV-VII was 1.8% (males 1.8%, females 1.8%, *p* = 0.896), and the prevalence of MetS remained stable during the observed period (*p* trends = 0.245). In both definitions, the prevalence of abdominal obesity and elevated FPG increased significantly, whereas the prevalence of low HDL-C decreased significantly. No significant changes were noted in the prevalence of elevated TG and elevated BP during KNHANES IV to VII. The overall trends in the prevalence of modified NCEP-ATP III MetS and its five components, according to KNHANES cycles by sex, are depicted in Figure 1. 

### 3.3. Prevalence of MetS According to Obesity Status 

The prevalence of MetS by modified NCEP-ATP III among normal-weight, overweight, and obese subjects was 1.3%, 8.2%, and 25.6%, respectively. The prevalence of MetS by IDF among normal weight, overweight, and obese was 0.0%, 1.9%, and 14.4%, respectively. The ORs of MetS by modified NCEP-ATP III in overweight subjects compared to the normal-weight subjects was 7.08 (95% CI, 5.19–9.79) and the ORs of MetS in obese subjects compared to normal-weight subjects was 27.13 (95% CI, 20.90–35.24). The ORs of MetS by IDF in overweight subjects compared to the normal-weight subjects was 42.29 (95% CI, 12.30–145.43) and the ORs of MetS in obese subjects compared to normal-weight subjects was 375.51 (95% CI, 119.29–1182.10). 

### 3.4. Diet Trends of the Study Subjects

**Figure 2:**
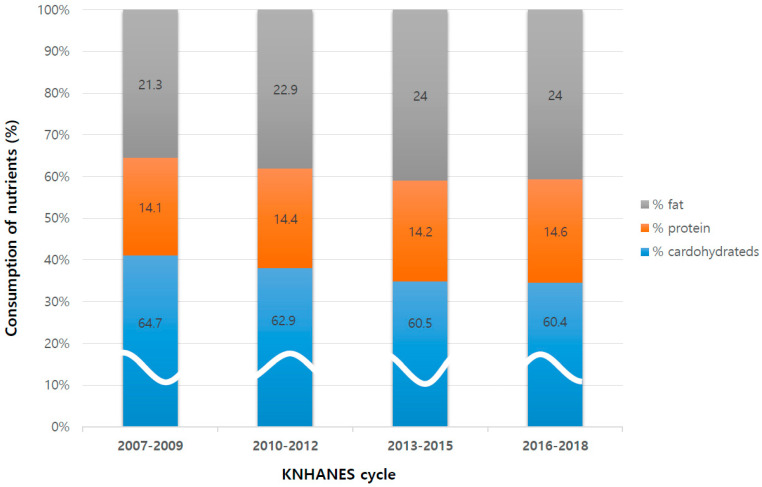
Overall trends in calorie consumption according to KNHANES cycles. There were trends of increasing protein and fat consumption. KNHANES, Korea National Health and Nutrition Examination Survey.

Table 3 and Figure 2 reports overall temporal trends in calorie consumption, including both macro-nutrients and sodium intake. Overall, male subjects had higher consumption of total calories, carbohydrate, protein, fat consumption, % protein, % fat consumption, and sodium intake. Female subjects had higher consumption of % carbohydrates, but there was no difference in % fat consumption. 

There were temporal trends of increasing total calorie consumption, increasing protein consumption, increasing fat consumption, and decreasing sodium intake. 

The percentage of total calories from carbohydrates significantly decreased over time (KNHANES-IV; 64.7% to VII; 60.4%, *p* < 0.001). The percentage of fat intake significantly increased over time (KNHANES-IV; 21.3% to VII; 24.0%, *p* < 0.001). The percentage of protein intake also increased over time (KNHANES-IV; 14.1.6% to VII; 14.6%, *p* = 0.029). 

Mean total calorie consumption was positively associated with systolic BP (*p* < 0.001), FPG (*p* = 0.001), and central obesity (*p* < 0.001) but inversely associated with HDL-C (*p* < 0.001) in total subjects. Carbohydrate intake was also positively associated with systolic BP (*p* < 0.001), FPG (*p* = 0.011), and central obesity (*p* < 0.001) but inversely associated with HDL-C (*p* < 0.001). There was no association between carbohydrate intake and log TG (*p* = 0.447). Protein intake was positively associated with systolic BP (*p* < 0.001), FPG (*p* < 0.001), and central obesity (*p* < 0.001), but inversely associated with HDL-C (*p* < 0.001). Fat intake was positively associated with systolic BP (*p* < 0.001), FPG (*p* = 0.011), and central obesity (*p* < 0.001). Only in females was fat intake was positively associated with HDL-C (*p* = 0.013). Sodium intake was positively associated with systolic BP (*p* < 0.001) and central obesity (*p* < 0.001) but inversely associated with HDL-C (*p* < 0.001). 

## 4. Discussion

In this study, using data of a national survey conducted in Korean children and adolescents aged 10–18 years, we found the current prevalence of MetS was 6.2% by modified NCEP-ATP III and 2.2% by IDF criteria. We also examined significant increasing trends in the prevalence of MetS, especially in male subjects between 2007 and 2018. The increased prevalence of obesity and hyperglycemia at this age group may be partly attributed to this phenomenon. 

It is difficult to estimate the prevalence of MetS in children because many different criteria have been used in its multiple definitions. Furthermore, different references and a cut-off value of its five components makes diagnosis more complicated [2]. In this study, the prevalence of MetS, going by modified NCEP-ATP III criteria in Korean children and adolescents increased, from 4.5% in 2007–2009 to 6.2% in 2016–2018. However, the prevalence of MetS by IDF criteria increased from 1.8% to 2.2% without significance. Further, each MetS component showed divergent trends. The central obesity and hyperglycemia significantly increased during that period, while low HDL-C showed considerably decreased trends. MetS prevalence using IDF criteria must include central obesity and meet two more risk factors. Since the frequency of low HDL-C is low and the criteria for the TG level are high, it is considered that there is no change in prevalence when IDF criteria are used.

In USA adolescents, the prevalence of MetS increased from 4.2% in NHANES 1988–1992 to 6.4% in NHANES 1999–2000 [15]. Although the MetS severity score was improved, the overall prevalence of ATP-III MetS from 1999 to 2012 was 9.83% [16]. Recently, the MetS prevalence ranged between 3.3% and 8.8% among U.S. adolescents for the years 2011–2016 depending on which MetS definition was used [17]. In China, the prevalence of MetS was 1.4% in 2013 [18]. Recently, the MetS prevalence increased to 2.3% in children aged 7–18 years from a Chinese National study with the same definition [19]. The trend of increasing obesity closely parallels that of increasing MetS. A review of several Korean studies revealed that the prevalence of MetS increased up to the early 2000s and began to decrease after the late 2000s [20,21,22]. However, they used different MetS along with different cut-offs from various references. On the contrary, our results show different results: the trend of MetS increased in Korean children and adolescents with the application of the same up to date criteria. 

The exact reason for increasing the prevalence of MetS is complex. Commonly, it was thought that the increasing prevalence of MetS seemed link to increasing rates of obesity, physical inactivity, and excessive nutrition in recent years. Obesity is hypothesized to be one of the predominant underlying causes of MetS; the interaction between obesity, insulin resistance, and inflammation plays a key role in its development [3,23]. In our study, the ORs of MetS by modified NCEP-ATP III in obese subjects compared to normal-weight subjects were 27.13 (95% CI, 20.90–35.24), which is consistent with the results of other studies [24,25]. Obesity also leads to impaired insulin signaling and subsequent insulin resistance. The amount of visceral fat is directly correlated with hyperinsulinemia and inversely correlated with insulin sensitivity [26]. Particularly, insulin resistance in the liver leads to decrease suppression of glucose production and results in hyperglycemia [27]. In our study, the prevalence of obesity increased from 8.4% to 10.6% and hyperglycemia from 5.3% to 12.0% during 2007–2018. These results were more pronounced in boys. In Korean adolescents, a positive linear trend is significant for diabetes (*p* trends = 0.048) and pre-diabetes (*p* trends < 0.001) after adjusting age [28]. In the modified NCEP-ATP III, abdominal obesity is a component, not a prerequisite. Especially in the case of boys, it is thought that the ORs increases due to a significant increase in hyperglycemia as obesity increases. Obesity among Asians is thought to be more harmful in terms of metabolic risk compared to other ethnic groups [29]. In Asian nations, the prevalence of impaired fasting glucose and diabetes in people with low BMI was relatively high and increased waist circumference was associated with this increased prevalence [30]. Thus, dietary interventions, especially calorie reduction, are primarily for obesity control. Gow et al. suggested that reduce the total energy of diet, irrespective of the macronutrient distribution, was successful in improving weight status in overweight and obese children and adolescents [31]. 

In this study, there were temporal trends of increasing total calorie, fat consumption during 2007 and 2018 in Korean children and adolescents. While calories from carbohydrates were stable and % of carbohydrates decreased. During the KNHANES cycle, mean HDL-C increased, resulting in a decrease in the prevalence of low HDL-C from 16.5% to 11.9%. In USA adolescents, both saturated fat and unsaturated fat intake were directly associated with HDL-C levels, and unsaturated fat was inversely associated with fasting TG levels [15]. Mensink et al. found that all types of fat ingestion, when substituted for carbohydrates, elevated HDL-C after reviewing 27 controlled trials [32]. Another meta-analysis examining the relationship between milk, fat and cardiovascular risk factors, found that those goods higher in saturated fat from whole milk and butter increased HDL-C when substituted for carbohydrates [33]. Finally, TC/HDL-C decreased by 0.31, 0.54, and 0.67 by replacing each 1% energy of total fat with saturated fat, monounsaturated fatty acids (MUFA) and polyunsaturated fatty acids (PUFA), respectively in controlled trials [34,35]. Thus, the substitution of good fat for carbohydrates is beneficially associated with HDL metabolism, while bad fat like trans-fat is significantly associated with a reduction of HDL-C. Since this lipoprotein has anti-atherogenic properties by reversing cholesterol transport from peripheral tissues to the liver, reducing calorie intake is more important than lowering carbohydrate levels.

The strengths of this study include the large representative sample of the Korean pediatric population surveyed, the use of standardized protocols, and data collection procedures through the KNHANE cycle. Although it is not a longitudinal study, the KNHANE gave us the trend of MetS and its components in children and adolescents. The present study also has several limitations. First, we could not show a significant trend to the prevalence of MetS using the IDF criteria. However, the components of MetS, including abdominal obesity and elevated FPG, showed significantly increases. Second, the present study was limited by its cross-sectional nature, which prevents our defining causal relationships between the trends of diet and MetS components. Third, we cannot distinguish the intake of unsaturated fat from saturated fat, although both fat intakes correlated with decreased prevalence of low HDL-C. Lastly, we could not assess the effects of subjects’ physical activity on MetS and its components. Physical activity is helpful in increasing HDL-C and decreasing both LDL-C and TG levels [36].

In conclusion, this study provided a recent survey of the prevalence of MetS in Korean children and adolescents. This study has importance in that it is nationally representative for the Korean population, is novel in its temporal assessment of MetS and its components, and explores possible lifestyle factors contributing to population health trends. Further studies are also needed to elucidate which lifestyle factors are involved in the increasing or decreasing trend of each MetS component. 

## Figures and Tables

**Table 1:** Characteristics of the study subjects according to the KNHANES cycle (2007–2018).

	KNHANES							
	IV–VII	IV	V	VI	VII	*p*-Value	b	*p*-Trend *
		2007–2009	2010–2012	2013–2015	2016–2018			
Number	8491	2549	2329	1800	1813			
Male (number)	4510	1344	1247	966	953			
Age (years)	13.8 ± 2.5	13.7 ± 2.5	13.7 ± 2.5	14.0 ± 2.5	14.0 ± 2.5	<0.001	0.047	<0.001
Height (cm)	160.4 ± 11.3	159.7 ± 11.6	160.2 ± 11.4	160.9 ± 10.9	161.0 ± 11.2	<0.001	0.046	<0.001
Weight (kg)	53.7 ± 14.0	52.7 ± 13.7	53.1 ± 13.8	55.1 ± 14.2	54.6 ± 14.4	<0.001	0.061	<0.001
Body mass index (kg/m^2^)	20.6 ± 3.7	20.4 ± 3.6	20.4 ± 3.6	21.0 ± 3.8	20.8 ± 3.8	<0.001	0.054	<0.001
Obesity status						0.032		0.002
BMI < 85th percentile	6711	2043	1865	1393	1410			
BMI ≥ 85th and < 90th percentile	862	267	222	189	184			
BMI ≥ 95th percentile	918	239	242	218	219			
Obesity (%)	10.8	9.3	10.4	12.1	12.1	<0.001		0.001
Waist circumference (cm)	69.4 ± 10.0	69.1 ± 9.7	68.7 ± 9.7	70.4 ± 10.1	69.9 ± 10.5	<0.001	0.046	<0.001
Fasting triglycerides (mg/dL)	85.8 ± 51.9	88.7 ± 55.2	82.4 ± 47.6	85.0 ± 52.5	87.0 ± 51.5	0.411	−0.821	0.412
HDL cholesterol (mg/dL)	50.9 ± 10.0	49.6 ± 9.5	51.0 ± 10.2	51.8 ± 10.0	51.7 ± 10.3	<0.001	0.082	<0.001
Systolic blood pressure (mmHg)	106.7 ± 10.2	105.1 ± 10.5	106.2 ± 10.3	108.4 ± 9.8	108.1 ± 9.8	<0.001	0.125	<0.001
Diastolic blood pressure (mmHg)	66.0 ± 9.1	66.3 ± 9.1	65.7 ± 9.6	65.9 ± 8.6	66.1 ± 8.9	0.583	−0.006	0.583
Fasting glucose (mg/dL)	90.2 ± 7.8	89.1 ± 6.6	89.1 ± 7.2	91.5 ± 9.2	91.8 ± 8.1	<0.001	0.151	<0.001

Data are means ± SD, number or percentage, HDL-C, High-density lipoprotein cholesterol; * Linear regression was used to estimate means of quantitative measurements. Logistic regression analyses were used for predicting the prevalence trends of MetS and its components.

**Table 2:** Trends and prevalence of metabolic syndrome and its components according to the KNHANES cycle (2007–2018).

				KNHANES			
	IV–VII	IV	V	VI	VII	*p*-Value	*p*-Trend
		2007–2009	2010–2012	2013–2015	2016–2018		
Number	8491	2549	2329	1800	1813		
Male (N)	4510	1344	1247	966	953		
**Modified NCEP-ATP III**							
Metabolic syndrome (%) *	4.6	4.5	3.9	4.1	6.2	0.002	0.020
Male	5.2	5.1	4.2	4.6	7.5	0.004	0.026
Female	3.9	3.8	3.5	3.5	4.8	0.456	0.346
Elevated FPG (%)	7.6	5.3	4.9	9.7	12.0	<0.001	<0.001
Low HDL-C (%)	13.1	16.5	12.5	10.1	11.9	<0.001	<0.001
TG level ≥ 110 mg/dL (%)	21.3	23.2	19.1	20.1	22.6	0.001	0.587
Elevated BP (%)	13.8	14.6	13.0	13.5	14.1	0.399	0.651
Central obesity (%)	9.2	8.4	8.2	10.1	10.6	0.016	0.004
**IDF**							
Metabolic syndrome (%) *	1.8	1.8	1.4	1.9	2.2	0.346	0.245
Male	1.8	1.6	1.4	2.1	2.4	0.240	0.077
Female	1.8	2.0	1.5	1.7	1.9	0.813	0.853
Elevated FPG (%)	7.6	5.3	4.9	9.7	12.0	<0.001	<0.001
Low HDL-C (%)	15.7	19.5	14.7	13.4	13.8	<0.001	<0.001
TG level ≥ 150 mg/dL (%)	8.7	9.6	7.9	7.9	9.0	0.106	0.401
Elevated BP (%)	3.4	3.3	2.9	3.8	3.6	0.408	0.368
Central obesity (%)	9.2	8.4	8.2	10.1	10.6	0.016	0.004

Data are numbers or percentages. Modified NCEP-ATP III definition of MetS: three of the following five risk factors: (1) FPG ≥ 100 mg/dL, (2) HDL-C level < 40 mg/dL, (3) TG level ≥ 110 mg/dL, (4) BP ≥ 90th percentile, and (5) waist circumference ≥ 90th percentile. * IDF definition of MetS: central obesity and two of the following four risk factors: (1) FPG ≥ 100 mg/dL, (2) HDL-C level < 40 mg/dL (if age > 16 years; <50 mg/dL), (3) TG level ≥ 150 mg/dL, (4) systolic BP ≥ 130 mmHg or a diastolic BP ≥ 85 mmHg.

**Table 3:** Diet trends of the study subjects across the KNHANES cycle (2007–2018).

				KNHANES				
	IV–VII	IV	V	VI	VII	*p*-Value	b	*p*-Trend
		2007–2009	2010–2012	2013–2015	2016–2018			
Number	7530	2229	2093	1653	1555			
Male (N)	3985	1177	1109	879	820			
Age (years)	13.8 ± 2.5	13.7 ± 2.5	13.7 ± 2.5	14.0 ± 2.5	14.0 ± 2.5			
Energy intake (kcal)	2098 ± 856	1938 ± 755	2181 ± 846	2194 ± 943	2112 ± 876	<0.001	60.7	<0.001
Carbohydrate (g)	321.8 ± 127.0	310.1 ± 120.2	337.1 ± 126.6	326.1 ± 134.8	313.5 ± 126.5	0.545	0.80	0.547
Protein (g)	75.5 ± 41.1	68.0 ± 31.5	79.1 ± 40.5	79.2 ± 50.2	77.7 ± 42.1	<0.001	3.21	<0.001
Fat (g)	55.2 ± 35.2	47.1 ± 29.0	57.6 ± 35.7	60.2 ± 38.1	58.1 ± 37.6	<0.001	3.84	<0.001
% of Carbohydrate (%)	62.4	64.7	62.9	60.5	60.4	<0.001	−1.565	<0.001
% of Protein (%)	14.3	14.1	14.4	14.2	14.6	<0.001	0.144	<0.001
% of Fat (%)	22.9	21.3	22.9	24.0	24.0	<0.001	0.947	<0.001
Water (g)	851 ± 492	777 ± 440	888 ± 488	968 ± 553	780 ± 472	0.003	15.04	0.003
Sodium (mg)	3724 ± 2177	3969 ± 2230	4102 ± 2286	3499 ± 2153	3103 ± 1772	<0.001	−300.1	<0.001

Data are means ± SD, number or percentage.
